# Virtual Reality Tour to Reduce Perioperative Anxiety in an Operating Setting Before Anesthesia: Randomized Clinical Trial

**DOI:** 10.2196/28018

**Published:** 2021-09-01

**Authors:** Lina Vogt, Martin Klasen, Rolf Rossaint, Ute Goeretz, Peter Ebus, Sasa Sopka

**Affiliations:** 1 AIXTRA Competence Center for Training and Patient Safety Medical Faculty RWTH Aachen University Aachen Germany; 2 Department of Anesthesiology Medical Faculty RWTH Aachen University Hospital Aachen Germany; 3 Faculty of Educational Science Open University Heerlen Netherlands

**Keywords:** anesthesia, anxiety, exposure, operating theater, patient empowerment, periperative trait anxiety, STOA, STAI, virtual reality, VR

## Abstract

**Background:**

Perioperative anxiety is a major burden to patients undergoing surgeries with general anesthesia.

**Objective:**

This study investigated whether a virtual operating room tour (VORT) before surgery can be used to ameliorate perioperative anxiety.

**Methods:**

We employed a randomized parallel-group design with 2 study arms to compare VORT to the standard operation preparation procedure. The study included 84 patients. A validated inventory (state-trait operation anxiety-state) was used to assess perioperative state anxiety before (T1) and after (T2) surgery. In addition, trait operation anxiety was evaluated with an additional validated inventory (state-trait operation anxiety-trait). Moreover, user ratings on the usefulness of VORT were assessed with an evaluation questionnaire. Study arms were compared for perioperative state anxiety with two-tailed independent samples *t* tests. Subjective ratings were correlated with STOA-Trait values to investigate possible associations between perioperative anxiety with perceived usefulness.

**Results:**

There were no significant differences in perioperative state anxiety between VORT and standard operation preparation procedures before and after the surgery. Nonetheless, patients’ ratings of VORT overall were positive. The tour was perceived as useful and, therefore, showed acceptance for VR use. These ratings were unrelated to the degree of perioperative anxiety.

**Conclusions:**

The subjective benefit of VORT could not be explained by a reduction of perioperative anxiety. Instead, VORT appears to serve the need for information and reduce uncertainty. In addition, VORT is perceived as beneficial regardless of the age of the patients. Considering this effect and the manageable organizational and financial effort toward implementation, the general use of VORT can be recommended.

**Trial Registration:**

ClinicalTrials.gov NCT04579354; https://clinicaltrials.gov/ct2/show/NCT04579354

## Introduction

Fear and anxiety are very common in patients prior to surgery with anesthesia. This perioperative anxiety can lead to additional discomfort, increased pain sensation, and increased stress symptoms [1,2]. It is a significant burden for patients and a potential reason for decreased compliance.

Virtual stimulus exposure is successful in exposure therapy for the treatment of anxiety and tension [3]. Patients are not confronted with real anxiety-inducing stimuli but with virtual representations of them [3]. The effectiveness of virtual stimulus exposure is well documented, especially for specific phobias [4-8], where it is not inferior to in vivo exposition [4]. Therefore, virtual stimulus exposure could also be suitable for minimizing operation-associated fears.

However, much less is known about the application of virtual reality (VR) exposure in reducing situational fear in people without anxiety disorders. The potential of VR exposure to reduce anxiety before surgery with general anesthesia has recently been explored with respect to perioperative anxiety. Ryu et al [9] applied a virtual operating room tour for 69 children 1 hour before surgery with general anesthesia, leading to lower preoperative anxiety and stress levels and higher compliance. However, data on the issue is sparse and inconsistent, especially when evaluating adults.

This study aimed to evaluate the use of VR exposure via a virtual operating room tour (VORT) on perioperative anxiety in adult patients undergoing surgeries with general anesthesia. Previous research has shown that perioperative anxiety exists both as a state (ie, the acute emotional or cognitive reaction of a patient before or after the operation) and as a trait (ie, the patient’s disposition to be more or less fearful about operations) [2]. We assumed that VORT reduced perioperative state anxiety in patients before and after surgery with general anesthesia. In addition to the exposure effect, information about anesthesia and treatment can be conveyed using VR at low cost and effort, thus strengthening individual health competence.

## Methods

### Ethics Approval

The study protocol was approved by the local Ethics Committee (EK 039/20). The study was designed and conducted in accordance with the latest version of the Declaration of Helsinki [10]. Moreover, the study was registered by clinicaltrials.gov on September 28, 2020 (NCT04579354).

### Participants

All participants were patients of the Clinic for Anesthesiology, RWTH Aachen University Hospital. The inclusion criteria included age over 18 years, elective surgery with general anesthesia, and a good knowledge of German. Exclusion criteria included emergency surgery, thoracic surgery, neurosurgery, and tumor surgery, as these can be associated with increased anxiety or inability to provide consent. All participants signed an informed consent form before participating in the study.

### Sample Size Planning

Sample size planning was based on a meta-analysis by Carl [4] on the effect of virtual exposure on anxiety. For comparisons of VR exposure with participants on a waiting list that receive treatment after the treatment groups, this study reported a mean effect size (Hedges's g) of 0.9, analogous to Cohen's *d*. Sample size planning using g multiplied by power for this effect size resulted in a sample size of 68 patients (34 per group) for a two-sided null hypothesis test (*t* test for independent samples), with a power of 0.95 and an α error probability of .05. To compensate for an expected dropout of 10%-15%, we targeted a total sample size of 80 patients (40 per group).

### Study Design

The study employed a randomized parallel-group design to compare VORT to the standard operation preparation procedure (STOPP). We employed the state and trait anxiety scales from the state-trait operation anxiety inventory (STOA) [2] as outcome variables. STOA is a validated inventory with good psychometric properties which assess operation-related trait and state anxiety on separate scales. Trait anxiety (STOA-T, 20 items) is the relatively stable disposition of a person to be anxious about surgeries, whereas state anxiety (STOA-S, 10 items) assesses acute fear reactions in situations shortly before or after surgery on 2 dimensions (cognitive anxiety and affective anxiety, 5 items each). Measuring a relatively stable trait, STOA-T was rated only before the surgery, whereas STOA-S was assessed before (T1) and after the surgery (T2). As an additional outcome, we employed an in-house designed questionnaire to assess the participants’ perception and evaluation of VORT after surgery. The questionnaire assessed different statements about VORT with 8 items on a 5-point Likert scale from 1 (do not agree) to 5 (strongly agree). Moreover, in 1 item, patients were asked to give VORT a school grade (according to the German grade system) between 1 (excellent) and 6 (very poor).

Participants were randomly assigned to one of the study arms (VORT vs STOPP) by a person not involved in data acquisition using the following procedure: for all patients (N=80), we created opaque assignment envelopes containing a folded paper with a study arm assignment (VORT or STOPP), with a total of 40 participants in each arm. The order of the envelopes was randomized before the beginning of the study. The assignment envelope for each patient was opened after these steps to avoid any bias in providing study information to the patient or the standardized explanatory interview. Thus, the assignment to the study arms was double-blind (to the study nurse or anesthetist and the patient) until after the standardized explanatory anesthesia interview. The procedural steps of the study can be described as follows:

#### Providing Study Information and Obtaining Informed Consent

Information was provided in written form, and the study nurse assisted the consent process by providing additional explanations and answered questions. Patients were informed about the study background, the study arms, and the content of the video. All patients received a paper copy of the information sheet. Informed consent was obtained in written form. This step was identical for all patients in both study arms (VORT and STOPP).

#### Standardized Explanatory Anaesthesia Interview

The interview consisted of a detailed explanation by the educating physician (anesthetist). A standardized information sheet was further used to explain the procedure and the anesthesia used, corresponding to the hospital's standard operating procedure, and it was identical for all patients in both study arms (VORT and STOPP).

#### Unblinding of the Study Arm Assignment

Unblinding entailed opening the assignment envelope and according assignment of the patient to one of the study arms.

#### Arm 1: Virtual Operation Room Tour (VORT)

Patients in this study arm subsequently watched a virtual tour of the operation room using Oculus Go Standalone VR (product number 301-00105-01; Facebook). The tour began with the evening conversation between the patient and a nurse about the approximate time of the operation and the desired sleeping medication. The next scene showed the morning conversation about premedication and drug administration. Furthermore, the route through the hospital from the normal ward to the holding area was illustrated. In the holding area, aspects of the “safe-surgery saves lives” checklist, such as the operation side (left or right), were simulated in the conversation between the patient and a nurse. Finally, a scene from the operating room was shown in which the actual preparations for anesthesia (ie, breathing tube and intravenous access) up to the induction of anesthesia were simulated.

The total duration of VORT was 6 minutes 28 seconds. VR videos were recorded with an Insta360Pro 3D camera (Arashi Vision Inc), with 4k resolution and an internal microphone. The video can be found online [11]. VR videos were recorded from a third-person perspective with a static camera mounted on a tripod. Early in the study, we alternatively considered using a video from a first-person perspective. The decision against the latter was based on three observations: (1) 360° recordings led to strange camera images when shot from the perspective of a person lying in bed, (2) first-person perspective might evoke a strange feeling of alienation (eg, in women), given the fact that the male actor’s voice was still audible, and (3) several pilot subjects reported simulation sickness when watching the first-person perspective video.

#### Arm 2: Standard Operation Preparation Procedure (STOPP)

Following the standard procedure of the hospital, patients in this study arm underwent no additional preparation.

#### Questionnaires T1 (Before Operation)

Assessment of STOA-T and STOA-S (T1). This step was identical for all patients in both study arms (VORT and STOPP).

#### Questionnaires T2 (After Operation)

The assessment of STOA-S (T2) for all patients was completed in both study arms within 48 hours after surgery. Simultaneously, patients in the VORT study arm completed the evaluation questionnaire.

### Research Hypothesis

1. H1: VORT reduces perioperative state anxiety before the surgery with general anesthesia compared to the standard procedure.
2. H2: VORT reduces perioperative state anxiety after the surgery with general anesthesia compared to the standard procedure.

Moreover, concerning the application of VORT, we were interested in its overall patient evaluation. Also, we wanted to explore the possibility of perceived usefulness, especially in patients with higher trait anxiety, in other words, if the VORT rating was associated with perioperative trait anxiety. Finally, we wanted to evaluate whether the evaluation of VORT depended on the patient’s age (eg, older patients’ limited experience with VR). Therefore, we additionally investigated the following explorative research questions:

1. ER1: How is the use of VORT prior to surgery evaluated and accepted by patients?
2. ER2: Is there a possible relationship between the evaluation of VORT and perioperative trait anxiety?
3. ER3: Is there a possible relationship between the evaluation of VORT and patients’ age?
4. ER4: Does VORT influence anesthesia-related STOA items?

### Data Analysis

Data were analyzed with SPSS Statistics (version 25; IBM). STOA-S sum scores for the two dimensions (cognitive and affective anxiety) were compared between study arms with two-tailed independent samples *t* tests separately for T1 (H1) and T2 (H2). To quantify agreement to the Likert scale items in the evaluation questionnaire (ER1), we calculated the median of the responses. Furthermore, we recoded the responses to disagreement (values 1 and 2), neutral (value 3), and agreement (values 4 and 5) and descriptively quantified the percentage of agreement. To evaluate a possible relationship between trait operation anxiety and the evaluation of VORT (ER2), we calculated Pearson correlations between the items from the evaluation questionnaire and STOA-T values. Finally, we calculated correlations between the items from the evaluation questionnaire and the patients’ age. A threshold of an α error probability of .05 (two-sided testing) was applied to all analyses.

## Results

### Overview

In total, 84 patients were included in the study. Dropouts (n=12; 14.3%) were in the expected range; however, since all dropouts occurred during the enrolment of the first 62 patients, we decided at this time point to create and randomize 20 additional assignment envelopes. The primary reason for dropouts was the early discharge of the patient. Other reasons included postponed or additional surgeries and postoperative complications. VORT-associated concerns or worries were not reported; therefore, VORT did not contribute to participation dropouts. One patient withdrew from study participation. Data from the remaining 72 patients (mean age 54.19, SD 15.94, with a range of 20 to 81 years), with 35 female and 37 male subjects, was analyzed (VORT: n=35 vs STOPP: n=37). Due to single missing data points, the numbers of included patients vary slightly for the different analyses. Results of the study are presented according to our hypotheses.

### H1: VORT Reduces Perioperative State Anxiety Before the Surgery as Compared to the Standard Procedure

No significant differences in state anxiety emerged between VORT and STOPP before surgery. This was the case for both the affective (n=71; VORT: mean 10.49, SD 4.61 vs STOPP: mean 10.83, SD 4.09; t_69_=–0.37; *P*=.74) and the cognitive dimension (n=67; VORT: mean 10.88, SD 4.32 vs STOPP: mean 10.70, SD 4.38; t_65_=–0.18; *P*=.86).

### H2: VORT Reduces Perioperative State Anxiety After the Surgery as Compared to the Standard Procedure

Similarly, no significant differences in state anxiety emerged between VORT and STOPP after the surgery. This was the case both for the affective (n=68; VORT: mean 8.53, SD 4.95 vs STOPP: mean 7.91, SD 3.26; t_66_=0.61; *P*=.55) and the cognitive dimension (n=71; VORT: mean 9.11, SD 4.19 vs STOPP: mean 9.17, SD 4.00; t_69_=–0.05; *P*=.96).

### ER1: How is the Use of VORT Prior To Surgery Evaluated by Patients?

The median values of the responses and proportion agreement to the statements are listed in Table 1. Altogether, the patients’ ratings of the VORT are positive. The results indicate that most patients perceived the VORT as helpful for preparation and can recommend it to other patients. A median school grade of 2 (corresponding to “good” in the German grade system) corresponds well with these ratings.

**Table 1:** Evaluation of the virtual operation tour.

Item	Median rating (1=do not agree; 5=strongly agree)	Agreement to statement (%)
The virtual operation room tour helped me to prepare for the surgery.	4	71.4
The information the operation room tour gave me was helpful for my preparation.	4	61.8
I found it helpful for my preparation to see the surgical environment before the surgery.	4	74.3
The virtual surgery tour calmed me down.	4	58.6
The virtual surgery tour was too detailed.	2	19.4
The virtual surgery tour was not detailed enough.	2	8.8
Should I be operated on again, I would like to do the virtual surgery tour again.	4	54.3
I can recommend the virtual surgery tour to other patients.	4	91.4

### ER2: Is There a Possible Relationship Between the Evaluation of VORT and Perioperative Trait Anxiety?

There were no significant correlations between any of the items from the evaluation of the VORT (Table 1) and STOA-T values of the patients (all *P*>.19).

### ER3: Is There a Possible Relationship Between the Evaluation of VORT and Patients’ Age?

There were no significant correlations between any of the items from the evaluation of the VORT (Table 1) and the age of the patients (all *P*>.10).

### ER4: Does VORT Have an Influence on Anesthesia-Related STOA Items?

Comparing VORT and STOPP with a two-tailed independent samples *t* test for all 7 items related to anesthesia treatment, the difference was only found in favor of VORT slightly below the predefined significance threshold (*P*=.047). Comparisons for all other items were not significant, resulting in no evidence that VORT addresses anesthesia-specific fear components.

Moreover, we calculated comparisons between men and women for all single VORT rating items in two ways to assure that men and women felt equally addressed by our video using a male protagonist. First, we calculated independent samples *t* tests for the Likert scale ratings. Second, we compared male and female participants using Χ^2^ tests to assess possible sex-dependent differences in the agreement to the statements (recoded ratings). For the *t* tests, no significant sex differences emerged concerning the ratings of VORT (all *P*>.17); for the Χ^2^ tests, we found one significant difference for item 6 (“the virtual surgery tour was not detailed enough”), with higher agreement among female participants (21.4%) compared to males (0%). Moreover, we compared male and female participants with respect to their overall rating of VORT (school grade) in an independent samples *t* test. Men and women did not differ in their school grades (*P*=.25), with women tending towards better grades (mean 1.86, SD 0.66) compared to men (mean 2.10, SD 0.54).

## Discussion

### VORT Effects

Although VORT did not affect perioperative anxiety, the tour received good patient ratings and was perceived as helpful for surgery preparation. Remarkably, the positive evaluation of the virtual surgery tour was independent of the fear of surgery (ie, the personality trait or trait anxiety). In other words, the subjective benefit of VORT for the patients could not be attributed to anxiety reduction. A possible explanation for this finding may be the beneficial effect of preoperative education and information. The assumption of uncertainty being a central element in the emergence of hospitalization stress was first raised by Mishel [12] and later specifically adapted to operation situations by Kagan and Bar-Tal [13], who reported that preoperative uncertainty negatively affected mental and physical recovery after surgery. This aligns with the findings of Moerman et al [14], showing that more than 80% of surgical patients had a positive attitude towards receiving information, with more than half of them having high information requirements. Receiving information about health care interventions positively affects several aspects of patients’ mental and physical health, such as recovery, pain levels, and psychological distress [15-17]. Thus, in our study, the positive ratings of VORT may be attributed to reducing uncertainty and satisfying the need for information. Similar to Moerman et al [14], we conclude that surgical fear and need for information are related but distinguishable factors and addressing either of them can reduce patients’ distress. For future studies, physiological measures such as heart rate, blood pressure, and skin conductivity may be relevant indicators for assessing the arousal of the patients. Including these physiological measures might help disentangle physiological processes and their cognitive appraisal [18]. For example, it seems conceivable that VORT evokes physiological arousal due to the operation-associated settings and situations but reduces subjective stress via cognitive control.

Surprisingly, VORT is perceived as beneficial irrespective of the patient’s age, indicating 2 interesting aspects. First, the need for information and the wish to reduce uncertainty seem independent of the patient’s age. Second, the acceptance of VORT was equally high among elder and younger patients. Therefore, there is no evidence that experience with VR may play any role in the evaluation or that elderly patients may feel uncomfortable with the VR setup. Given the high number of elderly patients undergoing surgeries with general anesthesia, these findings favor the general use of VORT in preoperative preparations.

Furthermore, there was no evidence that VORT addresses anesthesia-specific fear components. Our experience shows us that there are two patient groups: those who fear the anesthesia and its consequences in isolation and those who are only afraid of the procedure, the surgical part. As far as we know, no questionnaire provides information on fear of anesthesia in isolation.

### VR and Perioperative Anxiety

There is mixed evidence concerning the potential of VR in reducing perioperative anxiety. Some studies show no reduction in anxiety [19-21] by interventions as in our study, and others offer a detectable influence [22-26]. However, anxiety reduction has been applied preoperatively with good results, especially in children [9,26,27]. These inconsistencies may be partly attributed to the use of different anxiety inventories. The Spielberger State and Trait Anxiety Inventory (STAI) is frequently used [28]. However, the suitability of the STAI has been questioned for operation situations since it primarily measures anxiety in assessment situations [2]. Accordingly, more specific inventories have been developed, such as the STOA [2], the Amsterdam Perioperative Anxiety and Information Scale [14], and the Yale Preoperative Anxiety Scale [29], which was designed especially for children. The content of the items, length of the questionnaire, and other aspects vary considerably between these inventories, influencing study outcomes. For instance, Krohne et al [2] demonstrated that trait scores of STAI and STOA were correlated at r=.40, indicating that both inventories reflected a common factor of general anxiety but measured distinguishable facets of anxiety. Therefore, when comparing studies on VR interventions for perioperative anxiety, one must consider which inventories were used.

### Limitations

One possible limitation of our study is that all patients received the VR tour after the “educating interview.” Regarding patient compliance, it would be relevant to know whether there are fewer canceled surgeries when patients receive a VR education beforehand. Moreover, with the changed order, the VR tour could prepare patients for the anesthesia interview, enabling the patient to ask more detailed and specific questions. The second important limitation concerns the limited generalizability of our results to other types of anesthesia (eg, regional procedures). This is relevant in terms of distinguishing separate aspects of preoperative anxiety. Specifically, we assume that a distinction between fear of anesthesia and fear of the operation procedure by itself can be much more precise since several aspects of general anesthesia are not provided here (eg, fear of not waking up, lack of trust, or losing control while being unconscious).

Another limitation concerns whether the patients already had previous surgery with general anesthesia. Our study did not evaluate this aspect, and we cannot exclude the possibility it played a role in the VORT assessment. However, after careful consideration, we conclude that it is rather unlikely that prior surgical experience with anesthesia affected the VORT assessment. The probability of undergoing surgery increases with life age, suggesting that there was most likely a substantial correlation between life age and operations in the past in our study sample as well [30]. Our data found no correlation between life age and VORT ratings. We are aware the latter cannot distinguish between people who have experienced an operation and those who have not; however, we conclude that there is at least no indication from our data that having undergone an operation or not plays a role in the VORT rating.

### Conclusion

VORT is beneficial for a patient’s preparation for surgery with general anesthesia. Remarkably, this effect did not result in reduced perioperative anxiety. Instead, VORT seems to serve the need for information and to reduce uncertainty. Moreover, it is both accepted and recommended by patients. Considering the manageable organizational and financial effort and minimal time required in its implementation and the amount of time spent waiting in the perioperative outpatient clinic, the general use of VORT can be recommended.

## Appendix

Multimedia Appendix 1 CONSORT checklist.

## Abbreviations

STAI: Spielberger State and Trait Anxiety Inventory
STOA: state-trait operation anxiety
STOPP: standard operation preparation procedure
VORT: virtual operating room tour
VR: virtual reality
